# Neuroprotective Properties of Asiatic Acid against 5-Fluorouracil Chemotherapy in the Hippocampus in an Adult Rat Model

**DOI:** 10.3390/nu10081053

**Published:** 2018-08-09

**Authors:** Jariya Umka Welbat, Pornthip Chaisawang, Wanassanun Pannangrong, Peter Wigmore

**Affiliations:** 1 Department of Anatomy, Faculty of Medicine, Khon Kaen University, Khon Kaen 40002, Thailand; katainoy555@gmail.com (P.C.); wankun@kku.ac.th (W.P.); 2 Neuroscience Research and Development Group, Khon Kaen University, Khon Kaen 40002, Thailand; 3 School of Life Sciences, Medical School, Queen’s Medical Centre, Nottingham University, Nottingham NG7 2RD, UK; peter.wigmore@nottingham.ac.uk

**Keywords:** 5-fluorouracil, asiatic acid, neuroprotection, hippocampus

## Abstract

5-fluorouracil or 5-FU (a chemotherapeutic medication) has been revealed to induce memory deficits in many cancer patients. Asiatic acid (AA) is a triterpenoid extract from *Centella asiatica.* This compound can ameliorate intracellular oxidative stress caused by chemotherapy drugs. Recent studies have shown that AA is capable of inhibiting neuronal generation and memory deficit produced by 5-FU chemotherapy. This study aimed to assess the molecular mechanisms of AA related to hippocampal neurogenesis and memory in rats receiving 5-FU. Male Sprague Dawley rats were given AA (30 mg/kg) orally and given 5-FU (25 mg/kg) by i.v. injection 5 times. Some rats were given AA for 20 days before and during 15-FU treatment (preventive), some received AA for 20 days after 5-FU treatment (recovery), and some underwent treatment with AA throughout the time of the experiment (throughout) for 40 days. Treatment with 5-FU caused significant reductions in Notch1, sex determining region Y-box 2 (SOX2), nestin, doublecortin (DCX), and nuclear factor erythroid 2-related factor 2 (Nrf2) levels within the hippocampus. In addition, 5-FU significantly increased p21 positive cell number in the subgranular zone (SGZ) and malondialdehyde (MDA) levels in the hippocampus. Administration with both AA and 5-FU in prevention and throughout was able to prevent decreases in Notch1 SOX2, nestin, DCX, and Nrf2 caused by 5-FU. Treatment with AA also led to decreases in p21 positive cells and MDA levels in the hippocampus. These findings exhibit that AA has the ability to counteract the down-regulation of neurogenesis within the hippocampus and memory deficits caused by 5-FU via inhibiting oxidative stress and increasing neuroprotective properties.

## 1. Introduction

Chemotherapy is a principal treatment used to treat cancer patients. One of the possible side effects of chemotherapy is long-term memory impairment [1,2,3]. Moreover, chemotherapy remedy induces intracellular oxidative stress in both animals [4] and humans [5,6]. In addition, previous studies have found that chemotherapy drugs can enhance lipid peroxidation and upregulate levels of malondialdehyde (MDA) in the hippocampus [7,8,9]. Increases in MDA levels can cause cognitive impairment and neuronal loss in the hippocampus [10]. 5-fluorouracil (5-FU) is a chemotherapeutic medication and functions as an antimetabolite [11,12]. It is commonly utilized in the treatment of different types of cancers, such as bowel, prostate, and breast cancer [13]. It can passively diffuse across the blood brain barrier and is capable of damaging cell proliferation by inhibiting the enzyme thymidylate synthase, which is important for DNA replication [14,15]. 5-FU causes down-regulations in hippocampal cell division and survival and induces spatial working memory deficits [2,16,17]. However, 5-FU induced the memory dysfunction and reduction of hippocampal neurogenesis can be ameliorated by co-administration with fluoxetine [1,18]. 

Asiatic acid (AA) is a triterpenoid agent extracted from *Centella asiatica* (L.) Urban, which has many pharmacological activities, such as antioxidant and neuroprotective properties [19,20,21]. AA exhibits neuroprotective properties by acting as a cellular oxidative defense mechanism. 5-FU can cross the brain blood barrier (BBB) by simple diffusion [22] in animal studies. In humans, 5-FU can diffuse pass BBB and cause encephalopathy [23]. It has also been found to reduce blood pressure and MDA levels in a hypertensive rat model [24,25]. In addition, AA can protect against intracellular oxidative stress and reduce vital organ toxicity caused by chemotherapy drugs [26]. It has also been shown to improve learning and cognition, which are dependent on hippocampal neurogenesis in an animal model [27]. Furthermore, AA is found to have a preventive effect against hippocampal neurogenesis and spatial working memory impairment in rats given valproic acid [28] and 5-FU chemotherapy drugs [29]. The present study thus emphasizes the exploration of the molecular mechanisms of AA that are related to hippocampal neurogenesis and memory in rats receiving 5-FU. Oxidative stress was measured using the MDA assay and cell cycle arrest in the SGZ of the hippocampus was examined using p21 staining. Furthermore, Notch1, sex determining region Y-box 2 (SOX2), nestin, doublecortin (DCX), and nuclear factor erythroid 2-related factor 2 (Nrf2) levels were assessed using immunoblotting.

## 2. Materials and Methods

### 2.1. Animals and Treatments

Male Sprague Dawley rats (age 4–5 weeks, weight 180–220 g) were obtained from the National Laboratory Animal Center, Mahidol University, in Salaya, Nakornpathom, Thailand. Rats were habituated with a 12 h cycle of light and dark. They also had access to food and water ad libitum. All tests were done in accordance with the National Guidelines of Animal Care and were approved by the Animal Ethics Committee of Khon Kaen University (project number AEKKU 25/2557).

**Figure 1:**
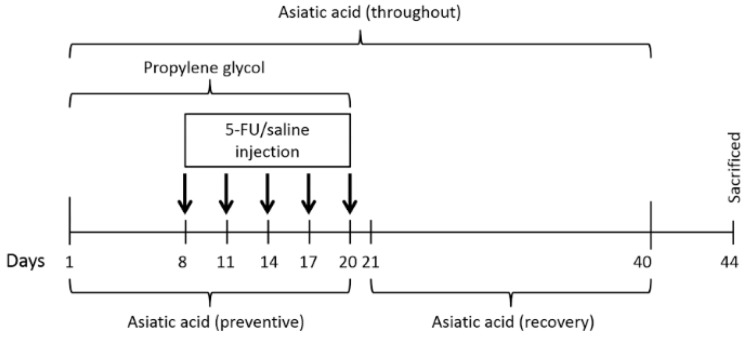
Timeline of animal drug administration. Single i.v. injections of 5-FU and saline are presented by thick arrows. The brackets show the period of time for propylene glycol and asiatic acid administration. 5-FU; 5-fluorouracil.

Sixty rats were randomly assigned into six groups (10 rats/group): control, 5-FU, AA, preventive, throughout and recovery groups. Rats in the control group were orally given propylene glycol (Ajax Finechem Pty Ltd., Auckland, New Zealand) not more than 1 mL/kg on day 1 to day 20 and received 0.9% sterile saline 5 intravenous (i.v.) injection. The 5-FU group treated with 5-FU (25 mg/kg, Boryung pharmaceutical Co., Ltd., Seoul, Korea) 5 times by i.v. injection, 3 days apart starting on day 8. Doses of 5-FU are within a range that could reduce tumor growth in rats [30]. The AA group received AA dissolved in propylene glycol (30 mg/kg, Faces Biochemical Co., Ltd., Wuhan, China) on day 1 to day 20. The preventive group was given AA on day 1 to day 20 and received 5-FU at an equal dose to the rats in 5-FU group. The throughout group was administered with 5-FU at an equal dose to the 5-FU group and received AA starting on day 1 to day 40. In recovery group, rats were administered with 5-FU (5 times by i.v. injection) at an equal dose to 5-FU group and started to receive AA on day 21 to day 40 (Figure 1). 

### 2.2. Tissue Preparation

All rats were euthanized by rapid stunning and cervical dislocation 3 days after the drug administration because it takes time to wash out of the body [31,32]. The brain was preserved in a cryoprotectant (30% sucrose solution) at 4 °C. Three hours after cryopreservation, the brain was snap frozen while embedded in Optimal Cutting Temperature (OCT) compound (Thermo Fisher Scientific, Karlsruhe, Germany) and stored at −80 °C for immunohistochemistry. The hippocampus was removed from the other half of the brain, snap-frozen rapidly in liquid nitrogen, and kept at −80 °C for immunoblotting and malondialdehyde (MDA) assay.

### 2.3. Immunohistochemistry

Cell cycle arrest was explored using p21 immuno-labeling. Frozen brains were cut at 40 μm thickness along the frontal plane using a freezing microtome (A.S. Science Co., Ltd., Walldorf, Germany). Sections were stored in a cryoprotective buffer at 4 °C. Nine sections were selected from every 8th section throughout the whole dentate gyrus. The sections were incubated with p21 primary antibody (Santa Cruz Biotechnology, Dallas, Texas, USA; sc-397; 1:100) for 24 h at 4 °C. Subsequently, they were incubated with goat anti-rabbit IgG secondary antibody (Alexa Fluor^®^568; Life Technologies, Carlsbad, CA, USA; A11011; 1:300) for 60 min and finally stained with DAPI (1:6000, Sigma Aldrich, Inc., St. Louis, MO, USA) for 30 s.

Eight sections were evaluated at X40 through a Nikon ECLIPSE 80i fluorescence microscope running NIS-Element AR 3.2 software (Melville, NY, USA). All p21 active cells within 3 cells from the innermost layer of the dentate gyrus were considered [27,28,29]. Summation of p21 active cell count in each hippocampus was multiplied by 8.

### 2.4. Western Blotting

The hippocampal tissue was homogenized to quantify protein expression as previously reported [2]. First, 45 µg of Notch1 and DCX proteins was loaded onto 10% and 12% SDS-polyacrylamide gels, respectively. Nestin levels were quantify by loading 20 µg of protein per lane onto 10% SDS-polyacrylamide gels. In addition, determination of SOX2 and Nrf2 levels was assessed by loading 20 µg of protein onto 12% SDS-polyacrylamide gels. Proteins were transferred onto nitrocellulose membranes. The blots were incubated overnight at 4 °C with primary antibodies as follows: polyclonal anti-Notch1 (Santa Cruz Biotechnology, Dallas, TX, USA; sc-6014; 1:100), polyclonal anti-DCX (Santa Cruz Biotechnology, Dallas, TX, USA; sc-8066; 1:150), monoclonal anti-nestin (Merck Millipore, MA, USA; MAB353; 1:1000), polyclone al anti-SOX2 (Abcam, Cambridge, UK; ab97959; 1:2000), polyclonal anti-Nrf2 (Abcam, Cambridge, UK; ab31163; 1:1000), and monoclonal mouse anti-GAPDH antibody (Abcam, Cambridge, UK; ab8245; 1:20,000). The blots were then washed and incubated with the secondary antibody (polyclonal goat anti-mouse; P0447, polyclonal goat anti-rabbit; P0448 and polyclonal rabbit anti-goat; P0449, Dako, Cambridge, UK; 1:2000). The blots were activated by an ECL solution (GE Healthcare, Buckingham, UK) and then measured protein density using ImageJ software (version 1.48 q). Data are presented as DCX (45 kilodalton; kDa), Notch1 (120 kDa), Nrf2 (68 kDa), SOX2 (34–40 kDa), and Nestin (200–220 kDa) optical density expressions as a ratio of GAPDH (36 kDa).

### 2.5. Assay of Malondialdehyde (MDA)

Thiobarbituric acid reactive substance (TBARS) was measured to show MMDA levels in the hippocampus. Tetraethoxypropane (TEP, Sigma Aldrich, Inc., St. Louis, MO, USA) was used as a standard solution. In brief, 100 μL of supernatant from tissue sample was mixed with 100 μL of 8.1% sodium dodecyl sulfate (Loba Chemie, Mumbai, India), 750 μL of 20% acetic acid solution (RCI Labscan, Bangkok, Thailand, pH 3.5), and 750 μL of a 0.8% thiobarbituric acid solution (TBA, Sigma Aldrich, Inc., St. Louis, MO, USA). The specimen was heated in a water bath at 95 °C for 60 min. The mixture was then centrifuged at 4000× *g* rpm for 10 min. The product of the TBA-MDA reaction was pink and had an absorbance of 540 nm according to spectrophotometric examination.

### 2.6. Statistical Analysis

All statistical parameters were calculated using GraphPad Prism version 5.0 and IBM SPSS Statistics version 17.0 (SPSS Inc., Chicago, IL, USA) and were presented as mean ± SEM. Statistical significance was assessed as *p* < 0.05. One-way ANOVA was used to evaluate a probability level of p21 positive cells, MDA levels, and protein expression. Least Significant Difference (LSD) was carried out to compare between groups when the results of one-way ANOVA were significant.

## 3. Results

### 3.1. Consequences of AA and 5-FU on the Expression of Notch1, SOX2, Nestin and DCX in the Hippocampus

**Figure 2:**
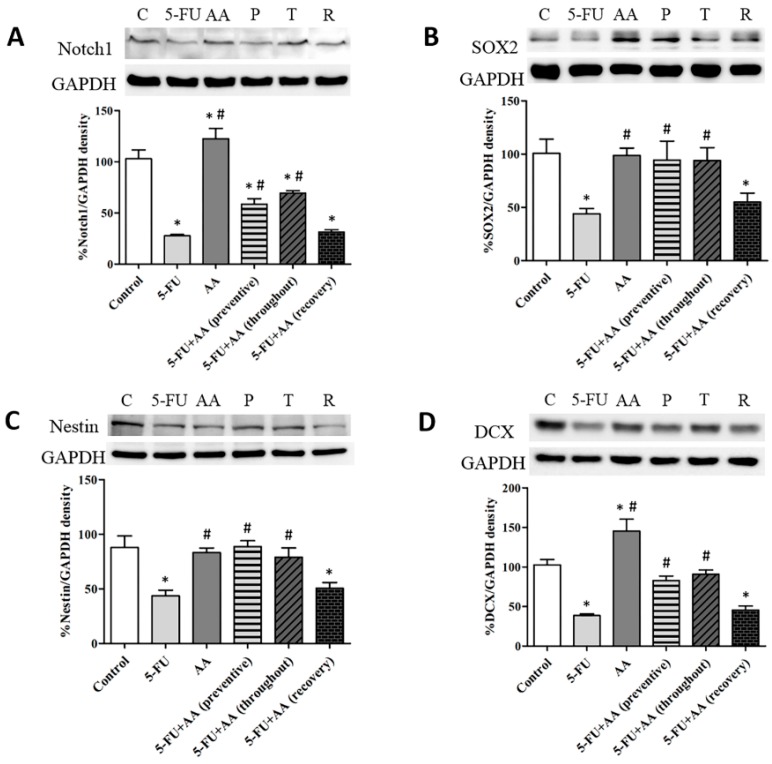
Mean ± SEM of Notch1 (**A**), sex determining region Y-box 2 or SOX2 (**B**), nestin (**C**) and doublecortin or DCX (**D**) protein expressions in the hippocampus were assessed by immunoblotting. * *p* < 0.05 compared with control group, # *p* < 0.05 compared with 5-FU group. GAPDH (glyceraldehyde 3-phosphate dehydrogenase), C; control group, AA; asiatic acid group, 5-FU; 5-fluorouracil group, P; preventive group, T; throughout group, R; recovery group.

Hippocampal Notch1, SOX2, nestin, and DCX expressions were determined via Western blotting (Figure 2). Examination of hippocampal Notch1 levels exhibited significant differences among the various groups (F_5,25_ = 49.96, *p* < 0.0001, Figure 2A). Notch1 levels in animals administered with 5-FU alone were significantly less than control, AA, preventive, and throughout rats (*p* < 0.05). Notch1 levels of rats received only AA were significantly greater than the control rats (*p* < 0.05). Moreover, Notch1 expressions in the rats received AA in recovery were not different from those in the rats treated with 5-FU (*p* > 0.05), but were significantly less than the control rats (*p* < 0.05). These results show that co-administration with AA in prevention or throughout the entire period of the experiment can improve hippocampal Notch1 protein expression. 

The examination of SOX2 protein expressions demonstrated a significant difference among the groups (F_5,30_ = 3.213, *p* = 0.0193, Figure 2B). Rats received 5-FU significantly expressed fewer SOX2 levels than the control, AA, or both 5-FU and AA (preventive and throughout) rats (*p* < 0.05). Rats in the recovery group did not show significant difference in SOX2 levels in comparison with the 5-FU rats (*p* > 0.05) but had significantly fewer SOX2 levels than the control rats (*p* < 0.05). These results suggest that AA is able to prevent decreases in SOX2 protein levels when administered before and during 5-FU treatments or throughout the entire treatment, but not after.

Nestin protein expression in the 5-FU treated group was significantly less than in the control group (F_5,30_ = 4.166, *p* = 0.0054, Figure 2C). Rats in the AA, preventive, and throughout groups showed nestin expressions significantly greater than the 5-FU group (*p* < 0.05). Surprisingly, nestin expressions in the recovery group were significantly less than those in control rats (*p* < 0.05). These results indicate that AA is potential to ameliorate decreases in nestin protein levels caused by 5-FU.

There were significant differences in terms of DCX levels among the various groups (F_5,25_ = 20.67, *p* < 0.0001, Figure 2D). Levels of DCX in the hippocampus in the rats given 5-FU significantly differed from those in the control, AA, preventive, and throughout groups (*p* < 0.05). Rats in the AA group showed a significant up-regulation of DCX levels when compared to the control group (*p* < 0.05). DCX protein expression in the recovery group did not significant differ from the 5-FU group (*p* > 0.05), but expression of this protein was significantly lower in the 5-FU group than in the control group (*p* > 0.05). These findings show that receiving AA in prevention or throughout the entire period of the experiment increased DCX protein expression to levels above those found in animals given 5-FU alone.

### 3.2. Effects of AA and 5-FU on the Expression of p21 in the Hippocampus

**Figure 3:**
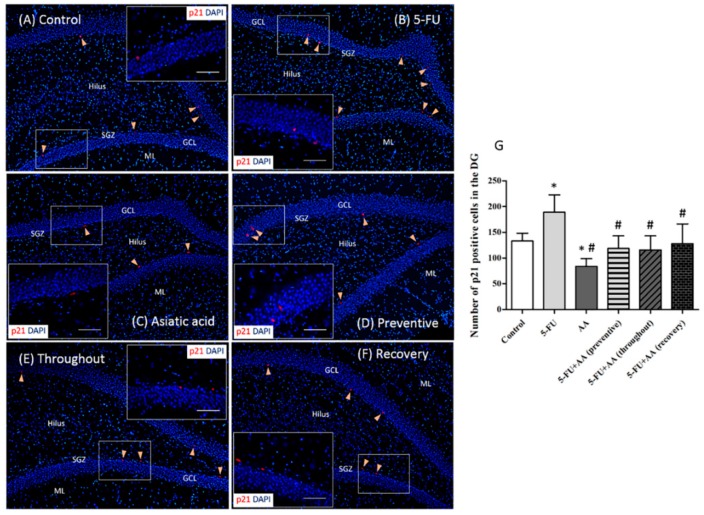
Images of p21 positive cells (red) in the dentate gyrus (**A**–**F**). All nuclei were stained with DAPI (blue). In the SGZ, p21 positive cells were indicated by arrowheads (scale bars 100 μm). p21 immunostaining was magnified as shown in the inserted images (scale bar 50 μm). p21 positive cell counts in the 5-FU group were significantly higher than those in the control, AA, preventive and throughout groups (*p* < 0.05, (**G**)). * *p* < 0.05 and # *p* < 0.05 compared with the controls and 5-FU groups, respectively. SGZ: subgranular zone, ML: molecular layer, GCL: granule cell layer.

Cell cycle arrest in the hippocampus was investigated using p21 (a marker of cell damage) immunostaining. p21 positive cell numbers in rats treated with only 5-FU were significantly higher than that in the controls (F_5,30_ = 9.783, *p* < 0.0001, Figure 3). Animals that received AA alone had significantly lower numbers of p21 positive cells when compared to the control and 5-FU rats (*p* < 0.05). In addition, the numbers of p21 positive cells in rats treated with AA were significantly less than those in the 5-FU group when rats received AA in prevention, recovery and throughout (*p* < 0.05). The data show that AA can inhibit the expression of p21 and repair cell damage caused by 5-FU.

### 3.3. Consequences of AA and 5-FU on Malondialdehyde (MDA) Levels in the Hippocampus

**Figure 4:**
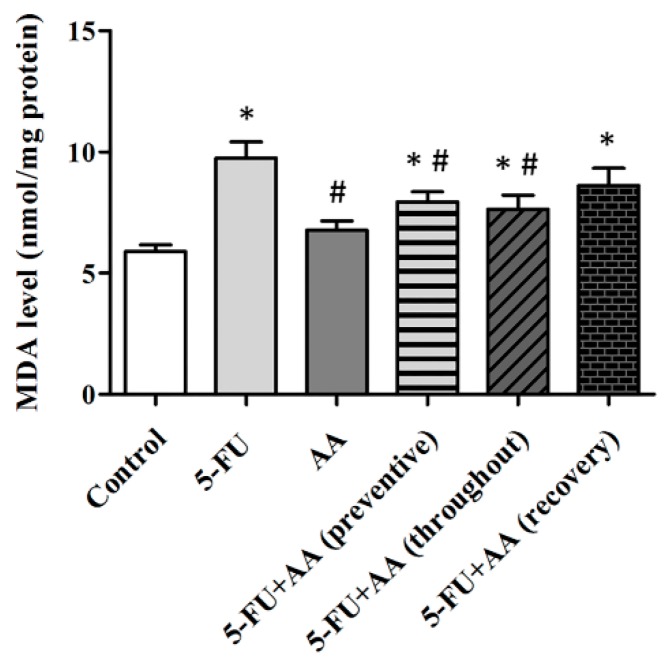
Malondialdehyde (MDA) levels (mean ± SEM) in the hippocampus. * *p* < 0.05 and # *p* < 0.05 compared with the controls and 5-FU groups, respectively. AA; asiatic acid, 5-FU; 5-fluorouracil.

Determination of MDA levels reveals that 5-FU per se were significantly higher than those in the control group (F_5,42_ = 6.568, *p* = 0.0001, Figure 4). Rats in the AA, preventive, and throughout groups showed significant decreases in MDA levels when compared to receiving only 5-FU (*p* < 0.05). Furthermore, MDA levels in the recovery group significantly differed from the control group (*p* < 0.05) but did not significantly differ from the 5-FU group. These findings suggest that receiving AA in prevention or throughout the whole duration of the experiment is able to counteract the impact of 5-FU on hippocampal MDA levels. 

### 3.4. Consequences of AA and 5-FU on Nrf2 Protein Expression in the Hippocampus

**Figure 5:**
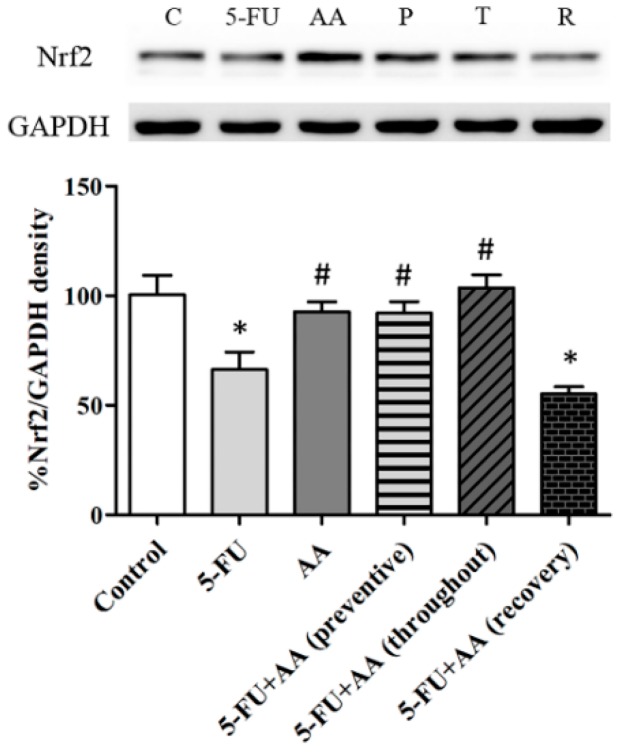
Mean ± SEM of nuclear factor erythroid 2-related factor 2 (Nrf2) protein in the hippocampus. * *p* < 0.05 and # *p* < 0.05 compared with the control and 5-FU groups, respectively. GAPDH (glyceraldehyde 3-phosphate dehydrogenase), C; control group, AA; asiatic acid group, 5-FU; 5-fluorouracil group, P; preventive group, T; throughout group, R; recovery group.

Western blots showed that the Nrf2 levels in the 5-FU group were significantly reduced in comparison with the control, AA, preventive, or throughout groups (F_5,36_ = 5.601, Figure 5). Additionally, the Nrf2 protein expression in the recovery group was significantly decreased when compared with the controls (*p* < 0.05). These results indicate that the Nrf2 protein levels of that are associated with antioxidant systems and hippocampal neurogenesis processes were up-regulated when AA was administered in prevention and the entire duration of the experiment.

## 4. Discussion

Newly generated neurons are important for adult brain plasticity and contribute to the functionality of hippocampal networks, which are required for learning and memory [33,34]. Many factors can influence neurogenesis process [35,36]. In the hippocampus, 5-FU chemotherapy treatment reduces cell division [2,16] and cell survival. It also induces spatial working memory deficits [1,29]. Moreover, neurogenesis in adult hippocampus is controlled by neurotransmitters, nerve growth factors, and transcription factors [37].

The present study postulates that 5-FU treatment decreased both Notch1 and DCX levels in the hippocampus. Notch1 (a transmembrane protein receptor) is found in neural stem cells in the hippocampus [38,39]. It is essential for proliferation and differentiation of neurons [40,41]. DCX (a microtubule associated protein) is mainly detected in immature neurons [42,43]. It is also needed for neuronal migration, differentiation, and plasticity [42,44]. These results confirm those of a previous report, in which 5-FU treatment was found to cause reductions in DCX protein levels in the hippocampus [2]. Previous studies have found that valproic acid (antiepileptic drug) treatment reduces Notch1 and DCX protein levels, leading to decreases in neurogenesis in the hippocampus [45]. Animals treated with AA alone exhibited significantly higher Notch1 and DCX expression than the control rats, a finding that is in line with those of our recent study [27]. Recent studies have found the ability of AA that prevent decreases in Notch1 caused by valproic acid in adult rats [28]. AA administration in prevention or throughout led to significantly higher Notch1 and DCX levels in comparison with rats treated with only 5-FU. Notch1 and DCX levels of animals treated with AA for 20 days after 5-FU treatment (recovery) did not return to control levels. 

SOX2 (a transcription factor) has a crucial function in cell division, self-renewal, and differentiation of neural stem cells [46,47]. Previous studies have reported that cisplatin (a chemotherapeutic drug) can decrease the number of SOX2 positive cells in the SGZ of the dentate gyrus and induce cognitive deficits in the rat brain [48]. Similarly, the present data show that 5-FU medication decreased hippocampal SOX2 expressions. In addition, animals treated with 5-FU alone had a significantly lower expression of nestin protein levels than the controls. Nestin (an intermediate filament protein) is important for self-renewal and survival of neural stem and progenitor cells [49,50]. These data are in similar to those from latest studies, which found suppression of nestin protein expression to be associated with decreases of neural progenitor cells (NPCs) induced by morpholino injection in zebrafish embryos [51]. In terms of SOX2 or nestin expression, no significant difference was found between rats received AA and the controls, indicating that AA did not enhance SOX2 or nestin expression in normal rats. However, SOX2 and nestin levels in rats received AA in prevention or throughout the entire duration of the experiment were significantly increased when compared to rats administered with 5-FU alone. By contrast, the expression SOX2 and nestin in animals given AA in recovery was not restored to control levels. These results indicate that AA may prevent the impairment of hippocampal neurogenesis enhanced by 5-FU agent by promoting Notch1, DCX, SOX2 and nestin protein expression in the hippocampus. However, these levels were not restored when AA was administered after chemotherapy.

Chemotherapy treatment can induce intracellular oxidative stress [5,52,53] and enhance oxidative damage of lipids and DNA. MDA is a major product of lipid peroxidation. In the present study, treatment with 5-FU chemotherapy increased MDA levels in the hippocampus. Similarly, cisplatin administration has been shown to significantly increase levels of MDA [9,10] and reduce the number of hippocampal neurons, changes which are concomitant with memory deficits [10]. Furthermore, previous studies have reported that 5-FU can induce expression of p21, a marker of cell damage, in cell culture [54]. Our study revealed that 5-FU per se significantly enhanced p21 positive cell numbers when compared to the controls. These findings demonstrate that 5-FU chemotherapy can cause hippocampal intracellular oxidative stress, which is concomitant with down-regulation of hippocampal neurogenesis.

There are various factors that can increase adult hippocampal neurogenesis including exercise, sleep, and the use of antidepressant drugs [55]. Previous studies have found that *Kaempferia parviflora* extract is capable of inhibiting memory deficits and reductions in hippocampal cell division caused by valproic acid [56]. Furthermore, co-administration with fluoxetine has been shown to ameliorate memory dysfunction and reductions in hippocampal cell generation found in 5-FU treatment [1,18]. Recent studies have found that AA can protect against memory impairment associated with cell proliferation and survival reductions in the SGZ of the hippocampus produced by 5-FU treatment [29]. AA also has antioxidant properties and protects against neuronal degeneration [21,57]. In addition, it can protect against intracellular oxidative stress and reduce liver [58], heart, and kidney tissue damage by increasing the Nrf2 expression [26]. Nrf2 has a crucial function in activating antioxidant mechanism and protecting against cell damage [59]. In addition, Nrf2 is essential for neuronal proliferation and differentiation in the hippocampus [60]. The present study found that treatment with 5-FU decreased Nrf2 protein expression. However, co-administration with AA before and during 5-FU treatments (preventive) or throughout the entire duration of the experiment (throughout) significantly increased the expression of Nrf2 and decreased p21 expression and MDA levels in the hippocampus. Co-administration of AA after (recovery) 5-FU treatment led to significantly lower levels of p21 when compared to administration of 5-FU alone. However, MDA and Nrf2 levels in rats administered AA in recovery were not different from the 5-FU rats. It is possible that AA enhances the antioxidant defense system and decreases lipid peroxidation and DNA damage if it is administered 5-FU in prevention and throughout but not in recovery. These results indicate that AA may prevent the reduction of hippocampal neuronal generation produced by 5-FU treatment by inhibiting intracellular oxidative stress.

## 5. Conclusions

In summary, increases of p21 (cell cycle arrest) and MDA (lipid peroxidation product) in the hippocampus produced by 5-FU was prevented by receiving AA. These results are associated with the increases of Notch1, SOX2, nestin, DCX, and Nrf2 expression in the hippocampus. However, it does not show a preventive effect of AA if the rats are given AA in recovery. Therefore, AA should be considered as an alternative drug for use in the prevention of cell damage in the hippocampus in patients taking 5-FU chemotherapy drugs.

## References

[1] Lyons L., ElBeltagy M., Bennett G., Wigmore P. (2012). Fluoxetine counteracts the cognitive and cellular effects of 5-fluorouracil in the rat hippocampus by a mechanism of prevention rather than recovery. PLoS ONE.

[2] Mustafa S., Walker A., Bennett G., Wigmore P.M. (2008). 5-fluorouracil chemotherapy affects spatial working memory and newborn neurons in the adult rat hippocampus. Eur. J. Neurosci..

[3] Myers J.S. (2009). Chemotherapy-related cognitive impairment. Clin. J. Oncol. Nurs..

[4] Khan R., Khan A.Q., Qamar W., Lateef A., Tahir M., Rehman M.U., Ali F., Sultana S. (2012). Chrysin protects against cisplatin-induced colon. Toxicity via amelioration of oxidative stress and apoptosis: Probable role of p38mapk and p53. Toxicol. Appl. Pharmacol..

[5] Amin K.A., Mohamed B.M., El-Wakil M.A., Ibrahem S.O. (2012). Impact of breast cancer and combination chemotherapy on oxidative stress, hepatic and cardiac markers. J. Breast Cancer.

[6] Il’yasova D., Kennedy K., Spasojevic I., Wang F., Tolun A.A., Base K., Young S.P., Kelly Marcom P., Marks J., Millington D.S. (2011). Individual responses to chemotherapy-induced oxidative stress. Breast Cancer Res. Treat..

[7] Ashok I., Sheeladevi R. (2014). Biochemical responses and mitochondrial mediated activation of apoptosis on long-term effect of aspartame in rat brain. Redox Biol..

[8] Helal G.K., Aleisa A.M., Helal O.K., Al-Rejaie S.S., Al-Yahya A.A., Al-Majed A.A., Al-Shabanah O.A. (2009). Metallothionein induction reduces caspase-3 activity and TNF alpha levels with preservation of cognitive function and intact hippocampal neurons in carmustine-treated rats. Oxid. Med. Cell. Longev..

[9] Jangra A., Kwatra M., Singh T., Pant R., Kushwah P., Ahmed S., Dwivedi D., Saroha B., Lahkar M. (2016). Edaravone alleviates cisplatin-induced neurobehavioral deficits via modulation of oxidative stress and inflammatory mediators in the rat hippocampus. Eur. J. Pharmacol..

[10] Chen C., Zhang H., Xu H., Zheng Y., Wu T., Lian Y. (2017). Ginsenoside Rb1 ameliorates cisplatin-induced learning and memory impairments. J. Ginseng Res..

[11] Lamson D.W., Brignall M.S. (1999). Antioxidants in cancer therapy; their actions and interactions with oncologic therapies. Altern. Med. Rev. J. Clin. Ther..

[12] Mihlon F.T., Ray C.E., Messersmith W. (2010). Chemotherapy agents: A primer for the interventional radiologist. Semin. Interv. Radiol..

[13] Fang L., Jiang Y., Yang Y., Zheng Y., Zheng J., Jiang H., Zhang S., Lin L., Zheng J., Zhang S. (2016). Determining the optimal 5-FU therapeutic dosage in the treatment of colorectal cancer patients. Oncotarget.

[14] Chu E., Callender M.A., Farrell M.P., Schmitz J.C. (2003). Thymidylate synthase inhibitors as anticancer agents: From bench to bedside. Cancer Chemother. Pharmacol..

[15] Longley D.B., Harkin D.P., Johnston P.G. (2003). 5-fluorouracil: Mechanisms of action and clinical strategies. Nat. Rev. Cancer.

[16] ELBeltagy M., Mustafa S., Umka J., Lyons L., Salman A., Dormon K., Allcock C., Bennett G., Wigmore P. (2012). The effect of 5-fluorouracil on the long term survival and proliferation of cells in the rat hippocampus. Brain Res. Bull..

[17] Wigmore P.M., Mustafa S., El-Beltagy M., Lyons L., Umka J., Bennett G. (2010). Effects of 5-FU. Adv. Exp. Med. Biol..

[18] ElBeltagy M., Mustafa S., Umka J., Lyons L., Salman A., Chur-yoe G.T., Bhalla N., Bennett G., Wigmore P.M. (2010). Fluoxetine improves the memory deficits caused by the chemotherapy agent 5-fluorouracil. Behav. Brain Res..

[19] Shukla A., Rasik A.M., Jain G.K., Shankar R., Kulshrestha D.K., Dhawan B.N. (1999). In vitro and in vivo wound healing activity of asiaticoside isolated from *Centella asiatica*. J. Ethnopharmacol..

[20] Huang S.S., Chiu C.S., Chen H.J., Hou W.C., Sheu M.J., Lin Y.C., Shie P.H., Huang G.J. (2011). Antinociceptive activities and the mechanisms of anti-inflammation of asiatic acid in mice. Evid.-Based Complement. Altern. Med..

[21] Krishnamurthy R.G., Senut M.C., Zemke D., Min J., Frenkel M.B., Greenberg E.J., Yu S.W., Ahn N., Goudreau J., Kassab M. (2009). Asiatic acid, a pentacyclic triterpene from *Centella asiatica*, is neuroprotective in a mouse model of focal cerebral ischemia. J. Neurosci. Res..

[22] Bourke R.S., West C.R., Chheda G., Tower D.B. (1973). Kinetics of entry and distribution of 5-fluorouracil in cerebrospinal fluid and brain following intravenous injection in a primate. Cancer Res..

[23] Formica V., Leary A., Cunningham D., Chua Y.J. (2006). 5-fluorouracil can cross brain-blood barrier and cause encephalopathy: Should we expect the same from capecitabine? A case report on capecitabine-induced central neurotoxicity progressing to coma. Cancer Chemother. Pharmacol..

[24] Bunbupha S., Pakdeechote P., Kukongviriyapan U., Prachaney P., Kukongviriyapan V. (2014). Asiatic acid reduces blood pressure by enhancing nitric oxide bioavailability with modulation of eNOS and p47^phox^ expression in l-NAME-induced hypertensive rats. Phytother. Res..

[25] Bunbupha S., Prachaney P., Kukongviriyapan U., Kukongviriyapan V., Welbat J.U., Pakdeechote P. (2015). Asiatic acid alleviates cardiovascular remodelling in rats with l-name-induced hypertension. Clin. Exp. Pharmacol. Physiol..

[26] Kamble S.M., Patil C.R. (2017). Asiatic acid ameliorates doxorubicin-induced cardiac and hepato-renal toxicities with Nrf2 transcriptional factor activation in rats. Cardiovasc. Toxicol..

[27] Sirichoat A., Chaijaroonkhanarak W., Prachaney P., Pannangrong W., Leksomboon R., Chaichun A., Wigmore P., Welbat J.U. (2015). Effects of asiatic acid on spatial working memory and cell proliferation in the adult rat hippocampus. Nutrients.

[28] Umka Welbat J., Sirichoat A., Chaijaroonkhanarak W., Prachaney P., Pannangrong W., Pakdeechote P., Sripanidkulchai B., Wigmore P. (2016). Asiatic acid prevents the deleterious effects of valproic acid on cognition and hippocampal cell proliferation and survival. Nutrients.

[29] Chaisawang P., Sirichoat A., Chaijaroonkhanarak W., Pannangrong W., Sripanidkulchai B., Wigmore P., Welbat J.U. (2017). Asiatic acid protects against cognitive deficits and reductions in cell proliferation and survival in the rat hippocampus caused by 5-fluorouracil chemotherapy. PLoS ONE.

[30] Watson S.A., Michael D., Justin T.A., Grimes S., Morris T.M., Robinson G., Clarke P.A., Hardcastle J.D. (1998). Pre-clinical evaluation of the gastrimmune immunogen alone and in combination with 5-fluorouracil/leucovorin in a rat colorectal cancer model. Int. J. Cancer.

[31] Miura K., Kinouchi M., Ishida K., Fujibuchi W., Naitoh T., Ogawa H., Ando T., Yazaki N., Watanabe K., Haneda S. (2010). 5-fu metabolism in cancer and orally-administrable 5-fu drugs. Cancers.

[32] Saif M.W., Syrigos K.N., Katirtzoglou N.A. (2009). S-1: A promising new oral fluoropyrimidine derivative. Expert Opin. Investig. Drugs.

[33] Gould E., Gross C.G. (2002). Neurogenesis in adult mammals: Some progress and problems. J. Neurosci..

[34] Ramirez-Amaya V., Marrone D.F., Gage F.H., Worley P.F., Barnes C.A. (2006). Integration of new neurons into functional neural networks. J. Neurosci..

[35] Ming G.L., Song H. (2011). Adult neurogenesis in the mammalian brain: Significant answers and significant questions. Neuron.

[36] Van Praag H., Christie B.R., Sejnowski T.J., Gage F.H. (1999). Running enhances neurogenesis, learning, and long-term potentiation in mice. Proc. Natl. Acad. Sci. USA.

[37] Balu D.T., Lucki I. (2009). Adult hippocampal neurogenesis: Regulation, functional implications, and contribution to disease pathology. Neurosci. Biobehav. Rev..

[38] Ables J.L., Breunig J.J., Eisch A.J., Rakic P. (2011). Not(ch) just development: Notch signalling in the adult brain. Nat. Rev. Neurosci..

[39] Pierfelice T., Alberi L., Gaiano N. (2011). Notch in the vertebrate nervous system: An old dog with new tricks. Neuron.

[40] Alberi L., Liu S., Wang Y., Badie R., Smith-Hicks C., Wu J., Pierfelice T.J., Abazyan B., Mattson M.P., Kuhl D. (2011). Activity-induced notch signaling in neurons requires Arc/Arg3.1 and is essential for synaptic plasticity in hippocampal networks. Neuron.

[41] Breunig J.J., Silbereis J., Vaccarino F.M., Sestan N., Rakic P. (2007). Notch regulates cell fate and dendrite morphology of newborn neurons in the postnatal dentate gyrus. Proc. Natl. Acad. Sci. USA.

[42] Manohar S., Paolone N.A., Bleichfeld M., Hayes S.H., Salvi R.J., Baizer J.S. (2012). Expression of doublecortin, a neuronal migration protein, in unipolar brush cells of the vestibulocerebellum and dorsal cochlear nucleus of the adult rat. Neuroscience.

[43] Rao M.S., Shetty A.K. (2004). Efficacy of doublecortin as a marker to analyse the absolute number and dendritic growth of newly generated neurons in the adult dentate gyrus. Eur. J. Neurosci..

[44] Wakabayashi T., Kosaka J., Mori T., Takamori Y., Yamada H. (2008). Doublecortin expression continues into adulthood in horizontal cells in the rat retina. Neurosci. Lett..

[45] Umka J., Mustafa S., ElBeltagy M., Thorpe A., Latif L., Bennett G., Wigmore P.M. (2010). Valproic acid reduces spatial working memory and cell proliferation in the hippocampus. Neuroscience.

[46] Julian L.M., Vandenbosch R., Pakenham C.A., Andrusiak M.G., Nguyen A.P., McClellan K.A., Svoboda D.S., Lagace D.C., Park D.S., Leone G. (2013). Opposing regulation of Sox2 by cell-cycle effectors E2f3a and E2f3b in neural stem cells. Cell Stem Cell.

[47] Sarkar A., Hochedlinger K. (2013). The sox family of transcription factors: Versatile regulators of stem and progenitor cell fate. Cell Stem cell.

[48] Lomeli N., Di K., Czerniawski J., Guzowski J.F., Bota D.A. (2017). Cisplatin-induced mitochondrial dysfunction is associated with impaired cognitive function in rats. Free Radic. Biol. Med..

[49] Rietze R.L., Valcanis H., Brooker G.F., Thomas T., Voss A.K., Bartlett P.F. (2001). Purification of a pluripotent neural stem cell from the adult mouse brain. Nature.

[50] Park D., Xiang A.P., Mao F.F., Zhang L., Di C.G., Liu X.M., Shao Y., Ma B.F., Lee J.H., Ha K.S. (2010). Nestin is required for the proper self-renewal of neural stem cells. Stem Cells.

[51] Chen H.L., Yuh C.H., Wu K.K. (2010). Nestin is essential for zebrafish brain and eye development through control of progenitor cell apoptosis. PLoS ONE.

[52] Lamberti M., Porto S., Marra M., Zappavigna S., Grimaldi A., Feola D., Pesce D., Naviglio S., Spina A., Sannolo N. (2012). 5-fluorouracil induces apoptosis in rat cardiocytes through intracellular oxidative stress. J. Exp. Clin. Cancer Res..

[53] Uzkeser H., Sener E., Bakan E., Hacimuftuoglu A. (2012). Preventive role of mirtazapine in methotrexate induced nephrotoxicity in rats. Scienceasia.

[54] Didelot C., Mirjolet J.F., Barberi-Heyob M., Ramacci C., Teiten M.H., Merlin J.L. (2003). Oncoprotein expression of E6 and E7 does not prevent 5-fluorouracil (5FU) mediated G(1)/S arrest and apoptosis in 5FU resistant carcinoma cell lines. Int. J. Oncol..

[55] Bruel-Jungerman E., Laroche S., Rampon C. (2005). New neurons in the dentate gyrus are involved in the expression of enhanced long-term memory following environmental enrichment. Eur. J. Neurosci..

[56] Welbat J.U., Chaisawang P., Chaijaroonkhanarak W., Prachaney P., Pannangrong W., Sripanidkulchai B., Wigmore P. (2016). Kaempferia parviflora extract ameliorates the cognitive impairments and the reduction in cell proliferation induced by valproic acid treatment in rats. Ann. Anat..

[57] Ramachandran V., Saravanan R. (2013). Efficacy of asiatic acid, a pentacyclic triterpene on attenuating the key enzymes activities of carbohydrate metabolism in streptozotocin-induced diabetic rats. Phytomedicine.

[58] Lv H., Qi Z., Wang S., Feng H., Deng X., Ci X. (2017). Asiatic acid exhibits anti-inflammatory and antioxidant activities against lipopolysaccharide and d-galactosamine-induced fulminant hepatic failure. Front. Immunol..

[59] Villeneuve N.F., Sun Z., Chen W., Zhang D.D. (2009). Nrf2 and p21 regulate the fine balance between life and death by controlling ROS levels. Cell Cycle.

[60] Karkkainen V., Pomeshchik Y., Savchenko E., Dhungana H., Kurronen A., Lehtonen S., Naumenko N., Tavi P., Levonen A.L., Yamamoto M. (2014). Nrf2 regulates neurogenesis and protects neural progenitor cells against Abeta toxicity. Stem Cells.

